# Supplementary material for: Inhibitory control and its modification in spider phobia – Study protocol for an antisaccade training trial
Source: PLoS One. 2023 Dec 19;18(12):e0292471. doi: 10.1371/journal.pone.0292471 (PMC10729957; doi:10.1371/journal.pone.0292471)
Supplement: S1 File — (PDF) [file pone.0292471.s002.pdf]

**Rat für Ethik in der Forschung (Ethikrat)**
**der Universität Siegen**

UNIVERSITÄT SIEGEN ● Ethikrat ● 57068 Siegen

Kontakt / Geschäftsstelle:
Frau Katrin Mayer (Dezernat 3)
Telefon: 0271 / 740-4819
E-Mail: ethikrat@uni-siegen.de

Aktenzeichen:

Siegen, 27.07.2021

**Project Description**

## 1. Name of the research project

Inhibitory control and its modification in the context of specific phobia

## 2. Name and contact details of the applicant(s) (office address)

Dr. Kati Roesmann

Institut für Psychologie

Adolf-Reichwein-Str. 2a

kati.roesmann@uni-siegen.de

+49 271 740-5123

**3. Information on the general conditions of the project.**

This is a pilot study within the framework of a mentoring program sponsored by the Division of Biological Psychology of the DGPs. A statement from the ethics committee is required.

**4. Subject and procedure of the project**

Anxiety is characterized by an attentional shift in favor of threatening stimuli[1], which according to the attentional control theory is caused by a reduced inhibition of bottom-up driven perceptual processes[2]. An impaired inhibitory control of attentional and perceptual processes due to anxiety could represent a risk factor, as well as a maintaining factor of pathological anxiety.

A paradigm for studying inhibitory control is the antisaccade paradigm[3], in which participants are asked to inhibit reflexive saccades toward visual stimuli presented in the peripheral field of view. Instead, an opposite (anti-)saccade to the mirror-image position should be performed[4]. Studies have shown that (subclinical) anxiety is associated with higher latencies in the execution of correct antisaccades, suggesting impaired inhibitory control as a psycho-physiological correlate of anxiety[5-9].

Due to the lack of studies on clinically relevant anxiety, the main aim of the present study is to compare a population of patients with spider phobia with a population of healthy control participants in terms of performance in the antisaccade paradigm. When performing the antisaccade paradigm, neutral visual stimuli as well as fear-specific stimuli (pictures of spiders) are examined. In order to investigate the role of impaired inhibitory control as a psycho-physiological correlate of (pathological) anxiety, further peripheral physiological measurements (see below) will also be performed in response to neutral and anxiety-specific stimuli. As a diagnostic procedure, in addition to questionnaires and an interview, a Behavioral Avoidance Test (BAT) will be performed, which involves a confrontation with a real tarantula (Theraphosidae). The corresponding data will be collected at the Department of Clinical Psychology Siegen in the context of a baseline measurement (see below for further explanation). The main relevant measure will be differences in antisaccade latencies and error rates between patients and participants. These will also be investigated for correlations with peripheral physiological measures.

Finally, pilot data will be collected in a proof-of-concept study (see below for more details) to examine whether a training of antisaccades in response to fear-specific stimuli can produce an improvement in antisaccade performance and whether this is accompanied by a change in BAT, as well as a change in peripheral physiological responses (see below) to fear-specific stimuli. Data on the modification of behavioral as well as peripheral physiological measures by means of an antisaccade training may provide clues regarding the potential for its application in a clinical context. This part of the study will also take place at the Department of Clinical Psychology on the same study date as the baseline measurement. The primary outcome of this part of the study represents the change in antisaccade latencies and error rates. These data will also be examined regarding correlations with the other measures collected (peripheral physiology, BAT, SPQ).

**Methods**

Diagnostic Methods:

- Structured Clinical Interview for DSM-5 (SCID-5)
- Spider-Phobia-Questionnaire (SPQ),
- Behavioral Avoidance Test (BAT): Standardized test to elicit avoidance behavior. Participants are asked to approach a real tarantula (Theraphosidae) in a plastic box as far as their fear allows. The final distance to the spider is used here as a quantification of spider fear at the behavioral level.
- Self-Assessment-Manikin (SAM): Graphically represented rating categories for evaluating valence and arousal.

Experimental Methods:

- Non-invasive recording of eye movements by means of eye tracking (device: Eye-Link 1000)
- Non-invasive derivation of skin conductivity via electrodes
- Non-invasive recording of heart rate via electrodes
- Repeated elicitation of the startle reflex by means of an acoustic stimulus (white noise, 50 ms, 105 dB(A), instantaneous rise time) and non-invasive recording of the muscular response via electrodes at the m. orbicularis oculi (unilateral)

**Experimental Tasks.**

Free-viewing-Paradigma:

The participants/patients are asked to look at various naturalistic pictures. These are eight neutral, eight negative affective, and eight fear-specific pictures (pictures of spiders). Each stimulus is presented three times. While viewing the images, heart rate, skin conductance, and startle reflex are recorded using the methods described above. Subsequently, the stimuli used are presented again so that the participants/patients can assess arousal and valence of the stimuli using the SAM. With a total of 72 trials, the duration of the paradigm is approximately 22.5 minutes.

Antisaccade-Task:

The participants/patients should first look at a fixation cross presented on a screen. As soon as a stimulus appears to the left or right of the fixation cross, the participants/patients are required to look to the mirror image position of the stimulus that has appeared to the side. The stimuli consist of affectively neutral stimuli (five schematic pictures of flowers) and fear-specific stimuli (five schematic pictures of spiders[10]). In addition to blocks requiring antisaccades, participants/patients are also asked at certain points to consciously look to the stimulus that appears to the side (i.e. to execute prosaccades). Throughout the task, participants'/patients' eye movements are recorded using the methodology described above. The paradigm contains a total of five blocks. Two blocks consisting of 60 prosaccades each and three blocks consisting of 40 antisaccades each. There is a one-minute pause between each block. The total duration of the paradigm is approximately 20 minutes.

Antisaccade-Training:

The participants/patients exclusively perform antisaccades in response to naturalistic spider images. Ten different stimuli are presented. The training takes place in two to three blocks of 80 runs each. The duration is approximately 15 minutes including a 5-minute break.

Prosaccade-Training:

Participants/patients perform exclusively prosaccades in response to naturalistic neutral images (e.g. of mushrooms). Ten different stimuli are presented. The training takes place in two to three blocks of 80 runs each. The duration is approximately 15 minutes including a 5-minute break.

**Procedure.**

Detailed description of the study procedure

Potential participants/patients are first contacted by telephone and screened for eligibility. In addition, diagnostic criteria of mental illnesses are checked by trained student personnel by means of the SCID in order to verify the presence of a specific phobia or the absence of a mental disorder. If the participants/patients are suitable and successfully diagnosed, they will be invited to the University of Siegen for actual study participation. Study participation includes a single on-site appointment, which includes baseline measurement, training, and post-measurement (only for patients with spider phobia). For healthy control participants, the total study duration comprises approximately four hours (including approximately three hours on-site). For patients with spider phobia, the total study duration comprises approximately five hours (including approximately four hours on-site).
This is followed by further diagnostics, which include the instruments described above (SPQ, BAT). After the diagnostics, the first execution of the free-viewing paradigm described above takes place. Following the free-viewing paradigm, the first implementation of the antisaccade paradigm takes place. Following the first execution of the antisaccade paradigm, participants /patients are randomly divided into two groups: One group (A) now undergoes the antisaccade training, while the other group (B) undergoes the prosaccade training. After the first training, the original antisaccade paradigm is performed again in order to investigate the influences of the training. In addition, a re-execution of the BAT takes place to quantify a possible decrease in avoidance behavior. After the BAT assessment, training conditions are switched for all patients with spider phobia. Group A now receives prosaccade training, while group B receives antisaccade training. Healthy control participants are no longer measured from this point on. Afterwards, a final execution of the antisaccade paradigm takes place, both for group A and for group B. After this, the free-viewing paradigm is performed again, as well as the BAT and the SPQ. After participation in the study, the patients will be paid in cash. Students of psychology may alternatively be compensated by the equivalent number of points for their courses.

**Physical impairment.**

The study may cause fatigue in the area of the eyes. Other physical stress is excluded.

The noise exposure due to the acoustic triggering of the startle reflex during the experiment is far below the valid limit values, which is why hearing damage is not to be expected in any case (cf.

BGV B3 (Noise) and corresponding EU Directive 2003/10/EC of February 6, 2003 "Minimum health and safety requirements regarding the exposure of workers to the risks arising from physical agents (noise)").

54 startle stimuli with an intensity of 105dB(A) and a duration of 50 ms each are presented. For comparison, even 200 stimuli at 105 dB per examination day would result in a dose/effect time of about 10 seconds. In relation to the permissible effective time of 4.8 minutes at 105dB(A), this value would be about 4% of the permissible noise exposure.

**Mental impairment.**

The participants/patients are confronted with negative affective images and fear-specific images (images of spiders). The stimulus material used has already been applied several times in studies, so that it is not to be expected that the stress caused by the negative images will lead to a long-term stress reaction in the test participants[11]. In addition, the confrontation with a live tarantula (Therphosidae) takes place. This can lead to increased aversive states (anxiety) in the short term, especially in the patients with spider phobia. However, these should not exceed the everyday level when confronted with spiders. Furthermore, the participants/patients will be accompanied by trained study personnel throughout the study and have the option to withdraw from the study at any time without giving reasons.

**Personal Information.**

Information on diagnostic criteria for mental disorders, as well as other somatic diagnoses and medication use, is needed.

**Deception.**

There is no deception.

**5. Information on recording, processing, storage and deletion of data.**

**Personal data.**

- Name
- Age

- Gender
- Address
- Medication
- Psychiatric diagnoses
- Somatic diagnoses

**Data protection.**

All data are collected and stored pseudonymously (i.e. with a study participant code). A subsequent allocation between person and own data can only be made with a data sheet, which is kept locked and destroyed after completion of the study (but after 10 years at the latest).

**Duty of confidentiality / obligation to maintain data secrecy.**

All employees of the study are participants to the obligation of secrecy.

**Retention and deletion of data.**

Data collected in the course of the study will be stored in a pseudonymized manner (i.e. coded without indication of name and address, or similar) on electronic data carriers. The data will be stored for 10 years in accordance with the applicable guidelines and then deleted.

**6. Obtaining the sample of persons and remuneration for participation.**

**Recruitment.**

Participants and patients with spider phobia should be recruited through participants appeals (e.g., flyers, newspaper ads, e-mail distribution lists).

**Participant sample from database?**

No existing sample from a database will be used.

**Characteristics of the sample.**

- Patients with spider phobia
- Healthy control participants

**Inclusion and exclusion criteria.**

<u>Inclusion</u>

- Age 18-65 years
- Current presence of a specific phobia (animal subtype: spider)
- visual acuity > 0.8
- normal hearing

<u>Exclusion:</u>

- Lifetime diagnosis of substance-related, bipolar, or psychotic disorder

- Presence of current mental disorder or history of mental disorder (except: current or lifetime mild-to-moderate depressive episode and specific phobia of animal subtype, if not predominant)
- Medication (benzodiazepines, barbiturates).
- Neurological disorders (esp. epilepsy)
- Organic mental disorders
- Dementia
- Injuries of the central nervous system
- Corneal deformity
- Hearing disorders (also tinnitus anamnestically), subjective hypersensitivity to sound (e.g. hyperacusis)
- regular nicotine consumption (> 5 cig. /day)

**Internet-based data collection.**

The questionnaire is conducted in digital form via LimeSurvey (https://www.limesurvey.org/de/) on site. This will ensure compliance with inclusion/exclusion criteria.

**Participation compensation.**

Participants/patients will receive compensation of 10€ per hour, which will be paid in cash. Students of psychology may alternatively receive points for their courses.

## 7. Voluntariness of participation and withdrawal

**Voluntariness.**

The voluntariness of participation is guaranteed by a comprehensive explanation, as well as special emphasis.

**Withdrawal.**

Ensuring the possibility of withdrawal at any time without disadvantages and the right to delete one's own data until the time of pseudonymization of the data.

## 8. Handling of abnormal findings

**Clarification.**

The results of the clinical interview are reported back to the test persons/patients on request. Otherwise, no findings will be collected whose feedback has relevance to the mental or physical health of the participants.

**Restriction on Participation.**

No restrictions on participation other than inclusion and exclusion criteria.

## 9. Information and consent.

### Information.

Full information of the participants/patients is ensured by a detailed explanation at the beginning of the study.

### Consent.

After participants have received and read the information, their consent is obtained (see Appendix).

### Image and sound recordings.

Image and sound recordings will not be collected.

## 10. Hygiene concept in the context of the Corona pandemic.

The study will be conducted under the Corona requirements and guidelines in effect at the time of implementation. In the sense of pandemic-resistant research, appropriate study elements (e.g. clinical diagnostics in the form of interviews) will be conducted digitally.

[…]

1. Mobini, S. & Grant, A. Clinical Implications of Attentional Bias in Anxiety Disorders: an Integrative Literature Review. *Psychotherapy* **44**, 450–462 (2007).
2. Eysenck, M. W., Derakshan, N., Santos, R. & Calvo, M. G. Anxiety and cognitive performance: Attentional control theory. *Emotion* **7**, 336–353 (2007).
3. Hutton, S. B. & Ettinger, U. The antisaccade task as a research tool in psychopathology: A critical review. *Psychophysiology* **43**, 302–313 (2006).
4. Hallett, P. E. Primary and secondary saccades to goals defined by instructions. *Vision Res.* **18**, 1279–1296 (1978).
5. Derakshan, N., Ansari, T. L., Hansard, M., Shoker, L. & Eysenck, M. W. Anxiety, inhibition, efficiency, and effectiveness: An investigation using the Antisaccade task. *Exp. Psychol.* **56**, 48–55 (2009).
6. Garner, M., Attwood, A., Baldwin, D. S., James, A. & Munafò, M. R. Inhalation of 7.5% carbon dioxide increases threat processing in humans. *Neuropsychopharmacology* **36**, 1557–1562 (2011).
7. Liang, C. W. Attentional control deficits in social anxiety: Investigating inhibition and shifting functions using a mixed antisaccade paradigm. *J. Behav. Ther. Exp. Psychiatry* **60**, 46–52 (2018).
8. Myles, O., Grafton, B. & MacLeod, C. Anxiety & inhibition: dissociating the involvement of state and trait anxiety in inhibitory control deficits observed on the anti-saccade task. *Cogn. Emot.* **34**, 1746–1752 (2020).
9. Basanovic, J. *et al.* Inhibitory attentional control in anxiety: Manipulating cognitive load in an antisaccade task. *PLoS One* **13**, 1–16 (2018).
10. Kolassa, I. T., Musial, F., Kolassa, S. & Miltner, W. H. R. Event-related potentials when identifying or color-naming threatening schematic stimuli in spider phobic and non-phobic individuals. *BMC Psychiatry* **6**, 1–12 (2006).
11. Dan-Glauser, E. S. & Scherer, K. R. The Geneva affective picture database (GAPED): A new 730-picture database focusing on valence and normative significance. *Behav. Res. Methods* **43**, 468–477 (2011).
