# Supplementary material for: Inhibitory control and its modification in spider phobia – Study protocol for an antisaccade training trial
Source: PLoS One. 2023 Dec 19;18(12):e0292471. doi: 10.1371/journal.pone.0292471 (PMC10729957; doi:10.1371/journal.pone.0292471)
Supplement: S2 File — (PDF) [file pone.0292471.s003.pdf]

Rat für Ethik in der Forschung (Ethikrat)
der Universität Siegen

UNIVERSITÄT SIEGEN ● Ethikrat ● 57068 Siegen

Kontakt / Geschäftsstelle:
Frau Katrin Mayer (Dezernat 3)
Telefon: 0271 / 740-4819
E-Mail: ethikrat@uni-siegen.de

Aktenzeichen:

Siegen, 27.07.2021

Vorhabensbeschreibung

1. Bezeichnung des Forschungsvorhabens

Inhibitionskontrolle und ihre Modulierbarkeit im Rahmen der spezifischen Phobie

2. Name und Kontaktdaten des/ der Antragstellenden (Dienstanschrift)

Dr. Kati Roesmann

Institut für Psychologie

Adolf-Reichwein-Str. 2a

kati.roesmann@uni-siegen.de

+49 271 740-5123

3. Angaben zu den Rahmenbedingungen des Vorhabens

Es handelt sich um eine Pilotstudie im Rahmen eines von der Fachgruppe für Biologische Psychologie der DGPs geförderten Mentoren-Programms. Eine Stellungnahme der Ethikkommission wird verlangt.

4. Gegenstand und Verfahren des Vorhabens

Gegenstand.

Angst ist durch eine Aufmerksamkeitsverschiebung zugunsten bedrohlicher Stimuli charakterisert[1], welche entsprechend der *Attentional-Control-Theory* durch eine verminderte Inhibition von *bottom-up* getriebenen Wahrnehmungsprozessen verursacht wird[2]. Eine durch Angst beeinträchtigte Inhibitionskontrolle von Aufmerksamkeits- und Wahrnehmungsprozessen könnte einen Risikofaktor, sowie einen aufrechterhaltenden Faktor von pathologischer Angst darstellen.

Ein Paradigma zur Untersuchung der Inhibitionskontrolle stellt das Antisakkadenparadigma dar[3], bei dem ProbandInnen reflexive Sakkaden in Richtung visueller Reize, die im peripheren Sichtfeld dargeboten werden, inhibieren sollen. Stattdessen soll eine entgegengesetzte (Anti-)Sakkade zur spiegelbildlichen Position ausgeführt werden[4]. Studien konnten zeigen, dass (subklinische) Ängstlichkeit mit höheren Latenzen bei der Ausführung korrekter Antisakkaden einhergeht, was eine beeinträchtigte Inhibitionskontrolle als psycho-physiologisches Korrelat von Ängstlichkeit nahelegt[5–9].

Aufgrund des Mangels an Studien zu klinisch relevanter Angst, besteht das Hauptziel der vorliegenden Studie im Vergleich einer Population von PatientInnen mit Spinnenphobie mit einer Population gesunder KontrollprobandInnen hinsichtlich der Performanz im Antisakkadenparadigma. Bei der Durchführung des Antisakkadenparadigmas werden sowohl neutrale visuelle Reize, als auch angstspezifische Reize (Bilder von Spinnen) untersucht. Um die Rolle einer beeinträchtigten Inhibitionskontrolle als psycho-physiologisches Korrelat von (pathologischer) Angst zu untersuchen, werden zudem weitere peripherphysiologische Messungen (s.u.) in Reaktion auf neutrale und angstspezifische Reize durchgeführt. Als diagnostisches Prozedere soll neben Fragebögen und eines Interviews auch ein *Behavioral Avoidance Test (BAT)* durchgeführt werden, bei dem es zu einer Konfrontation mit einer lebendigen Vogelspinne (*Theraphosidae)* kommt. Die entsprechenden Daten sollen am Lehrstuhl für klinische Psychologie Siegen im Rahmen einer Baseline-Messung (nähere Erläuterung s.u.) erhoben werden. Als hauptsächlich relevantes Maß sind hier Unterschiede bzgl. Antisakkaden-Latenzen und –Fehlerraten zwischen PatientInnen und ProbandInnen zu nennen. Diese sollen zudem auf Zusammenhänge mit peripherphysiologischen Maßen hin untersucht werden.

Abschließend sollen in einer proof-of-concept Untersuchung (nähere Erläuterung s.u.) Pilotdaten erhoben werden, die prüfen, ob das Training von Antisakkaden als Reaktion auf angstspezifische Reize eine Verbesserung der Antisakkaden-Performanz hervorrufen kann und ob diese mit einer Veränderung im BAT, sowie einer Veränderung peripherphysiologischer Reaktionen (s.u.) auf angstspezifische Reize einhergeht. Daten zur Modulierbarkeit von Verhaltens- sowie peripherphysiologischen Maßen durch das Antisakkadentraining kann Hinweise auf das Potential einer Anwendung im klinischen Kontext geben. Dieser Teil der Studie findet ebenfalls am

Lehrstuhl für klinische Psychologie zum selben Studientermin wie die Baseline-Messung statt. Das *primary-outcome* dieses Studienteils stellt die Veränderung von Antisakkaden-Latenzen und –Fehlerraten dar. Diese Daten werden ebenfalls auf Zusammenhänge mit den anderen erhobenen Maßen (Peripherphysiologie, BAT, SPQ) untersucht.

Methoden.

Diagnostische Methoden:

- Structured Clinical Interview for DSM-5 (SCID-5), Deutsche Version: Strukturiertes klinisches Interview zur Diagnosestellung psychischer Erkrankungen.
- Spider-Phobia-Questionnaire (SPQ), Deutsche Version: Fragebogen zur Erhebung der Symptomschwere der Spinnenangst.
- Behavioral Avoidance Test (BAT): Standardisierter Test zur Erhebung von Vermeidungsverhalten. Die Versuchspersonen werden gebeten sich einer lebendigen Vogelspinne (*Theraphosidae)* in einer Plastikbox anzunähern, soweit es ihnen ihre Angst erlaubt. Die finale Distanz zur Spinne wird hierbei als Quantifizierung der Spinnenängstlichkeit auf behavioraler Ebene genutzt.
- Self-Assessment-Manikin (SAM): Graphisch dargestellte Bewertungskategorien zur Evaluation von Valenz und Arousal.

Experimentelle Methoden:

- Non-invasive Erfassung von Augenbewegungen mittels Eye-Tracking (Gerät: Eye-Link 1000)
- Non-invasive Ableitung der Hautleitfähigkeit über Elektroden
- Non-invasive Ableitung der Herzrate über Elektroden
- Wiederholte Elizitation des Schreckreflexes mittels eines akustischen Reizes (weißes Rauschen, 50 ms, 105 dB(A), instantaneous rise time) und non-invasive Ableitung der muskulären Antwort über Elektroden am m. orbicularis oculi (unilateral)

Experimentelle Aufgaben.

Free-viewing-Paradigma:

Die ProbandInnen/PatientInnen sollen verschiedene naturalistische Bilder betrachten. Dabei handelt es sich um acht neutrale, acht negativ affektive, sowie acht angstspezifische Bilder (Bilder von Spinnen). Jeder Stimulus wird dreimal angeboten. Während der Betrachtung der Bilder werden Herzrate, Hautleitfähigkeit und Schreckreflex mit den oben beschriebenen Methoden erfasst. Im Anschluss werden die verwendeten Stimuli erneut dargeboten, sodass die ProbandInnen/PatientInnen Arousal und Valenz der Stimuli mittels des *SAM* beurteilen können. Bei insgesamt 72 Trials beträgt die Dauer des Paradigmas ca. 22,5 Minuten.

Antisakkaden-Paradigma:

Die ProbandInnen/PatientInnen sollen zunächst ein auf einem Bildschirm dargebotenes Fixationskreuz betrachten. Sobald ein links oder rechts vom Fixationskreuz dargebotener Stimulus erscheint sollen die ProbandInnen/PatientInnen zur spiegelbildlichen Position des seitlich aufgetretenen Stimulus blicken. Die Stimuli bestehen aus affektiv neutralen Reizen (fünf schematische Bilder von Blumen) und angstspezifischen Reizen (fünf schematische Bilder von Spinnen[10]). Konträr zu den Antisakkaden werden die ProbandInnen/PatientInnen zudem an bestimmten Stellen aufgefordert bewusst zum seitlich auftauchenden Stimulus zu blicken (Prosakkade). Während der gesamten Aufgabe werden die Augenbewegungen der ProbandInnen/PatientInnen mittels der oben beschriebenen Methodik aufgezeichnet. Das Paradigma enthält insgesamt fünf Blöcke. Zwei Blöcke bestehend aus jeweils 60 Prosakkaden und drei Blöcke bestehend aus jeweils 40 Antisakkaden. Zwischen den Blöcken erfolgt jeweils eine Minute Pause. Die Gesamtdauer des Paradigmas beträgt ca. 20 Minuten.

Antisakkaden-Training:

Die ProbandInnen/PatientInnen führen ausschließlich Antisakkaden in Reaktion auf naturalistische Spinnenbilder aus. Dabei werden zehn verschiedene Stimuli präsentiert. Das Training erfolgt in zwei bis drei Blöcken mit jeweils 80 Durchgängen. Die Dauer beträgt inklusive einer 5-minütigen Pause ca. 15 Minuten.

Prosakkaden-Training:

Die ProbandInnen/PatientInnen führen ausschließlich Prosakkaden in Reaktion auf naturalistische neutrale Bilder (z.B. von Pilzen) aus. Dabei werden zehn verschiedene Stimuli präsentiert. Das Training erfolgt in zwei bis drei Blöcken mit jeweils 80 Durchgängen. Die Dauer beträgt inklusive einer 5-minütigen Pause ca. 15 Minuten.

Durchführung.

Ablauf Baseline-Messung:

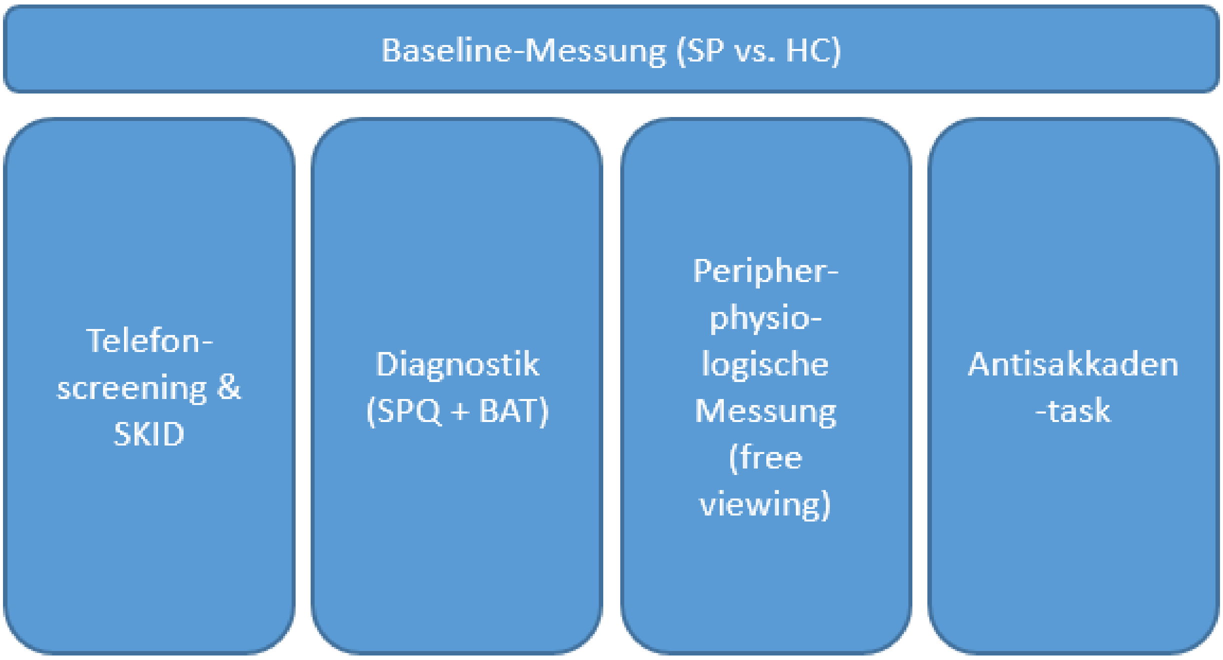

Abbildung 1 Schematische Abbildung der Baseline-Messung. SP = spider phobics. HC = healthy controls. SKID = Strukturiertes klinisches Interview DSM-5. SPQ = Spider Phobia Questionnaire. BAT = Behavioral Avoidance Test.

Ablauf Training und Post-Messung:

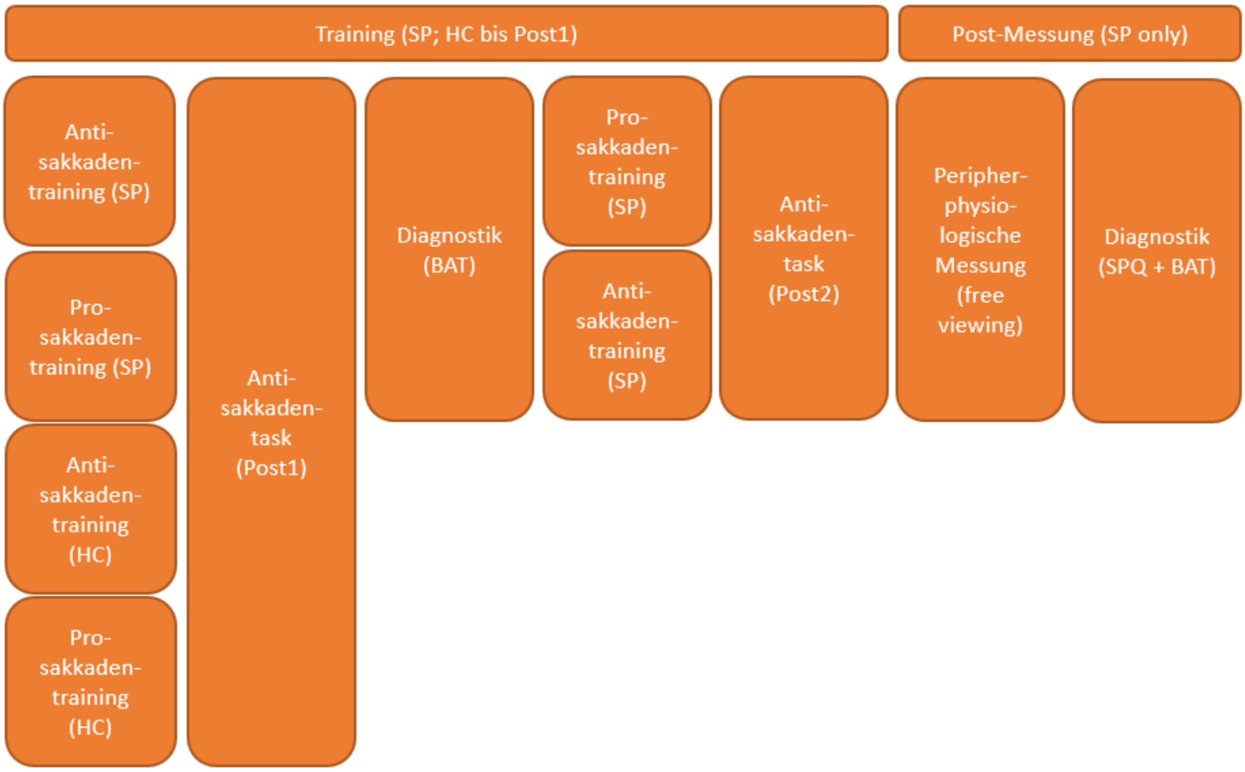

Abbildung 2 Schematische Abbildung der proof-of-concept Komponente. SP = spider phobics. HC = healthy controls. SPQ = Spider Phobia Questionnaire. BAT = Behavioral Avoidance Test.

Detaillierte Beschreibung des Studienprocedere:

Potentielle ProbandInnen/PatientInnen werden zunächst telefonisch kontaktiert und mittels eines Screenings auf Eligibilität geprüft. Telefonisch werden zudem diagnostische Kriterien psychischer Erkrankungen mittels des *SCID-5* durch geschultes studentisches Personal geprüft, um das Vorliegen einer spezifischen Phobie, bzw. das Nicht-Vorliegen einer psychischen Störung zu verifizieren. Bei Eignung und erfolgreicher diagnostischer Zuordnung der ProbandInnen/PatientInnen werden diese zur eigentlichen Studienteilnahme an die Universität Siegen eingeladen. Die Studienteilnahme umfasst einen einzelnen Termin vor Ort, welcher Baseline Messung, Training und Post-Messung (nur für PatientInnen mit Spinnenphobie) umfasst. Für gesunde KontrollprobandInnen umfasst die Gesamtstudiendauer ca. vier Stunden (davon ca. drei Stunden vor Ort). Für PatientInnen mit Spinnenphobie umfasst die Gesamtstudiendauer ca. fünf Stunden (davon ca. vier Stunden vor Ort).

Zu Beginn des Termins erfolgt zunächst erneut eine ausführliche schriftliche und mündliche Aufklärung der ProbandInnen/PatientInnen (siehe Anhang), sowie das Ausfüllen einer Einverständniserklärung (siehe Anhang).

Danach erfolgt eine weitergehende Diagnostik, welche die oben beschriebenen Instrumente (*SPQ, BAT*) umfasst.

Nach der Diagnostik erfolgt die erste Durchführung des oben beschriebenen free-viewing-Paradigmas.

Im Anschluss an das free-viewing-Paradigma erfolgt die erste Durchführung des Antisakkaden-Paradigmas.

Im Anschluss an die erste Durchführung des Antisakkaden-Paradigmas werden ProbandInnen/PatientInnen randomisiert in zwei Gruppen unterteilt: Die eine Gruppe (A) durchläuft nun das Antisakkaden-Training, während die andere Gruppe (B) das Prosakkaden-Training durchführt.

Nach dem ersten Training erfolgt nun eine erneute Durchführung des ursprünglichen Antisakkaden-Paradigmas, um Einflüsse des Trainings zu untersuchen. Zudem erfolgt eine erneute Durchführung des *BAT*, um eine mögliche Abnahme von Vermeidungsverhalten zu quantifizieren.

Nach der erneuten Durchführung des *BAT* erhält nun die jeweils andere Gruppe der PatientInnen jeweils das Antisakkaden-Training oder Prosakkaden-Training. Gruppe A erhält ein Prosakkaden-Training, während nun Gruppe B ein Antisakkaden-Training erhält. Gesunde KontrollprobandInnen werden ab diesem Zeitpunkt nicht mehr gemessen.

Im Anschluss erfolgt eine letzte Durchführung des Antisakkaden-Paradigmas, sowohl für Gruppe A als auch für Gruppe B.

Hiernach erfolgt eine erneute Durchführung des free-viewing-Paradigmas, sowie eine erneute Durchführung des *BAT*, sowie erneute Erhebung des *SPQ.*

Nach Teilnahme an der Studie wird den PatientInnen die entsprechende Vergütung in bar ausgezahlt. StudentInnen der Psychologie können sich alternativ durch die äquivalente Anzahl an Versuchspersonenstunden vergüten lassen.

Körperliche Beanspruchung.

Die Studie kann Ermüdungserscheinungen im Bereich der Augen hervorrufen. Andere körperliche Beanspruchung ist ausgeschlossen.

Die Lärmexposition durch die akustische Auslösung des Schreckreflexes während des Experiments liegt weit unterhalb der gültigen Grenzwerte, weshalb in keinem Falle Gehörschädigungen zu erwarten sind (vgl. BGV B3 (Lärm) und entsprechenden EU-Richtlinie 2003/10/EG vom 6. Februar 2003 "Mindestvorschriften zum Schutz von Sicherheit und Gesundheit der Arbeitnehmer vor der Gefährdung durch physikalische Einwirkungen (Lärm)").

Es werden 54 Schreckreize mit einer Intensität von 105dB(A) und einer Dauer von jeweils 50 ms präsentiert. Zum Vergleich: Selbst bei 200 Stimuli mit 105 dB pro Untersuchungstag würde eine Dosis/Wirkzeit von etwa 10 Sekunden erreicht. In Bezug auf die zulässige Wirkzeit von 4,8 Minuten bei 105dB(A) würde dieser Wert ca. 4 % der zulässigen Lärmbelastung betragen.

Mentale Beanspruchung.

Die ProbandInnen/PatientInnen werden mir negativ affektiven Bildern, sowie angstspezifischen Bildern (Bildern von Spinnen) konfrontiert. Das verwendete Stimulusmaterial hat dabei schon

mehrfach in Studien Anwendung gefunden, wodurch nicht zu erwarten ist, dass der durch die negativen Bilder hervorgerufene Stress bei den Versuchspersonen zu einer längerfristigen Stressreaktion führt[11]. Zudem erfolgt die Konfrontation mit einer lebendigen Vogelspinne *(Therphosidae).* Dies kann kurzfristig insbesondere bei den PatientInnen mit Spinnenphobie zu verstärkten aversiven Zuständen (Angst) führen. Diese sollten jedoch nicht über das alltägliche Maß bei Konfrontation mit Spinnen hinausgehen. Die ProbandInnen/PatientInnen werden zudem die ganze Zeit von geschultem Studienpersonal begleitet und haben die Möglichkeit die Studie jederzeit ohne Angabe von Gründen abzubrechen.

Preisgabe persönlicher Informationen.

Informationen zu diagnostischen Kriterien psychischer Störungen, sowie zu sonstigen somatischen Diagnosen und Medikamenteneinnahme werden benötigt.

Täuschung und Aufklärung.

Es wird ohne Täuschung gearbeitet.

5. Angaben zu Aufzeichnung, Aufbereitung, Speicherung und Löschung der Daten

Personenbezogene Daten.

- Name
- Alter
- Geschlecht
- Adresse
- Medikamenteneinnahme
- Psychiatrische Diagnosen
- Somatische Diagnosen

Datenschutz.

Alle Daten werden pseudonymisiert erhoben und abgespeichert (d.h. mit einem Versuchspersonencode). Eine nachträgliche Zuordnung zwischen Person und eigenen Daten kann nur mit einem Datenblatt erfolgen, welches verschlossen aufbewahrt und nach Abschluss der Studie (spätestens aber nach 10 Jahren) vernichtet wird.

Schweigepflicht / Verpflichtung auf das Datengeheimnis / Verschwiegenheit.

Alle Mitarbeitenden der Studie unterliegen der Schweigepflicht.

Aufbewahrung und Löschung der Daten.

Im Rahmen der Studie erhobene Daten werden pseudonymisiert (d.h. kodiert ohne Angabe von Namen und Anschrift, o.ä.) auf elektronischen Datenträgern gespeichert. Die Daten werden nach den geltenden Richtlinien 10 Jahre gespeichert und anschließend gelöscht.

6. Gewinnung der Personenstichprobe und Teilnahmevergütung

Rekrutierung.

ProbandInnen und PatientInnen mit Spinnenphobie sollen durch Probandenaufrufe (z.B. Flyer, Zeitungsanzeigen, E-Mailverteiler) rekrutiert werden.

Personenstichprobe aus Datenbank?

Es soll keine bestehende Personenstichprobe aus einer Datenbank genutzt werden.

Merkmale der Personenstichprobe.

- PatientInnen mit Spinnenphobie
- Gesunde KontrollprobandInnen

Einschluss- und Ausschlusskriterien.

Einschluss:

- Alter 18-65 Jahre
- Aktuelles Vorliegen einer spezifischen Phobie (Tier-Subtypus: Spinne)
- Visus > 0.8
- Normalhörigkeit

Ausschluss:

- Lebenszeit-Diagnose einer substanzbezogenen, bipolaren oder psychotischen Störung
- Vorliegen einer aktuellen psychischen Störung oder einer psychischen Störung in der Vergangenheit (außer: aktuelle oder lifetime leichte bis mittelgradige depressive Episode und spezifische Phobie vom Tiertypus, sofern diese nicht im Vordergrund stehen)
- Medikation (Benzodiazepine, Barbiturate)
- Neurologische Erkrankungen (insbes. Epilepsie)
- Organische psychische Störungen
- Demenz
- Verletzungen des zentralen Nervensystems
- Hornhautverkrümmung
- Hörstörungen (auch Tinnitus anamnestisch), subjektive Geräuschüberempfindlichkeit (z.B. Hyperakusis)
- regelmäßiger Nikotinkonsum (> 5 Zig. /Tag)

Internetbasierte Datengewinnung.

Der Fragebogen wird in digitaler Form via LimeSurvey (https://www.limesurvey.org/de/) Vorort durchgeführt. Dadurch wird die Einhaltung der Ein-/Ausschluss-Kriterien sichergestellt.

Teilnahmevergütung.

Die ProbandInnen/PatientInnen erhalten eine Vergütung in Höhe von 10€ pro Stunde, welche bar ausgezahlt wird. StudentInnen der Psychologie können alternativ Versuchspersonenstunden erhalten.

7. Freiwilligkeit der Teilnahme und Rücktritt

Freiwilligkeit.

Die Freiwilligkeit der Teilnahme wird durch eine umfassende Aufklärung, sowie besondere Betonung gewährleistet.

Rücktritt.

Sicherstellung der jederzeitigen Rücktrittsmöglichkeit ohne Nachteile und des Rechts auf Löschung der eigenen Daten bis zum Zeitpunkt der Pseudonymisierung der Daten.

8. Umgang mit auffälligen Befunden

Aufklärung.

Die Ergebnisse des klinischen Interviews werden den ProbandInnen/PatientInnen auf Rückfrage zurückgemeldet. Ansonsten werden keine Befunde erhoben, deren Rückmeldung eine Relevanz für die psychische oder körperliche Gesundheit der Teilnehmenden haben.

Teilnahmebeschränkung.

Keine Teilnahmebeschränkungen neben Ein- und Ausschlusskriterien.

9. Informiertheit und Einwilligung

Informiertheit.

Die vollständige Informiertheit der ProbandInnen/PatientInnen ist durch eine ausführliche Aufklärung zu Beginn der Studie gewährleistet.

Einwilligung.

Nachdem die Teilnehmenden die Information erhalten und gelesen haben, wird ihre Einwilligung eingeholt (siehe Anhang).

Bild- und Tonaufnahmen.

Bild- und Tonaufnahmen werden nicht erhoben.

10. Hygienekonzept im Rahmen der Corona-Pandemie

Die Studie wird unter den zum Zeitpunkt der Durchführung geltenden Corona-Auflagen und Vorgaben durchgeführt. Im Sinne einer *pandemic-resistant*-Forschung werden entsprechende Studienelemente (z.B. klinische Diagnostik in Form von Interviews) digital durchgeführt.

[…]

1. Mobini, S. & Grant, A. Clinical Implications of Attentional Bias in Anxiety Disorders: an Integrative Literature Review. *Psychotherapy* **44**, 450–462 (2007).

2. Eysenck, M. W., Derakshan, N., Santos, R. & Calvo, M. G. Anxiety and cognitive performance: Attentional control theory. *Emotion* **7**, 336–353 (2007).

3. Hutton, S. B. & Ettinger, U. The antisaccade task as a research tool in psychopathology: A critical review. *Psychophysiology* **43**, 302–313 (2006).

4. Hallett, P. E. Primary and secondary saccades to goals defined by instructions. *Vision Res.* **18**, 1279–1296 (1978).

5. Derakshan, N., Ansari, T. L., Hansard, M., Shoker, L. & Eysenck, M. W. Anxiety, inhibition, efficiency, and effectiveness: An investigation using the Antisaccade task. *Exp. Psychol.* **56**, 48–55 (2009).

6. Garner, M., Attwood, A., Baldwin, D. S., James, A. & Munafò, M. R. Inhalation of 7.5% carbon dioxide increases threat processing in humans. *Neuropsychopharmacology* **36**, 1557–1562 (2011).

7. Liang, C. W. Attentional control deficits in social anxiety: Investigating inhibition and shifting functions using a mixed antisaccade paradigm. *J. Behav. Ther. Exp. Psychiatry* **60**, 46–52 (2018).

8. Myles, O., Grafton, B. & MacLeod, C. Anxiety & inhibition: dissociating the involvement of state and trait anxiety in inhibitory control deficits observed on the anti-saccade task. *Cogn. Emot.* **34**, 1746–1752 (2020).

9. Basanovic, J. *et al.* Inhibitory attentional control in anxiety: Manipulating cognitive load in an antisaccade task. *PLoS One* **13**, 1–16 (2018).

10. Kolassa, I. T., Musial, F., Kolassa, S. & Miltner, W. H. R. Event-related potentials when identifying or color-naming threatening schematic stimuli in spider phobic and non-phobic individuals. *BMC Psychiatry* **6**, 1–12 (2006).

11. Dan-Glauser, E. S. & Scherer, K. R. The Geneva affective picture database (GAPED): A new 730-picture database focusing on valence and normative significance. *Behav. Res. Methods* **43**, 468–477 (2011).
